# The Global Economic Impact of Manta Ray Watching Tourism

**DOI:** 10.1371/journal.pone.0065051

**Published:** 2013-05-31

**Authors:** Mary P. O’Malley, Katie Lee-Brooks, Hannah B. Medd

**Affiliations:** 1 WildAid, San Francisco, California, United States of America; 2 The Manta Trust, Dorchester, United Kingdom; 3 Shark Savers, New York, New York, United States of America; National Institute of Water & Atmospheric Research, New Zealand

## Abstract

As manta rays face increased threats from targeted and bycatch fisheries, manta ray watching tourism, if managed properly, may present an attractive economic alternative to consumptive use of these species. Both species in the genus Manta (Manta alfredi and Manta birostris) are classified by the International Union for the Conservation of Nature Red List as species Vulnerable to extinction in the wild, and are considered unsustainable as fisheries resources due to their conservative life history characteristics, which considerably reduce their ability to recover population numbers when depleted. Utilising dive operator surveys, Internet research, and a literature review, this study provides the first global estimate of the direct economic impact of manta ray watching tourism and examines the potential socio-economic benefits of non-consumptive manta ray watching operations relative to consumptive use of manta rays as a fishery resource. In the 23 countries in which manta ray watching operations meeting our criteria were identified, we estimated direct revenue to dive operators from manta ray dives and snorkels at over US$73 million annually and direct economic impact, including associated tourism expenditures, of US$140 million annually. Ten countries account for almost 93% of the global revenue estimate, specifically Japan, Indonesia, the Maldives, Mozambique, Thailand, Australia, Mexico, United States, Federated States of Micronesia and Palau. In many of the areas where directed fisheries for manta rays are known to occur, these activities overlap with manta ray tourism sites or the migratory range of the mantas on which these sites depend, and are likely to be unsustainable and detrimental to manta ray watching tourism.

## Introduction

While some conservation biologists assert that the intrinsic value of nature should provide sufficient ethical justification for its conservation [1], [2], [3], environmental policy decision makers are nevertheless challenged with balancing the needs of numerous stakeholders amid increasing competition for the use of valuable and diminishing natural resources [4]. Accordingly, interest in exploring the benefits of marine recreational activities as non-consumptive uses of marine resources has grown considerably in recent years [5]–[7]. One such non-consumptive option is ecotourism, which can be defined as “non-consumptive travel with minimal negative impact that results in increased conservation and sustainability of natural and sociocultural resources and contributes to the well-being of local people” [8]. Ecotourism in the marine realm, focused on large marine species (megafauna), can, if properly managed, potentially offer one solution that provides long-term, sustainable benefits for both the people and animals involved [9], [10].

Marine species involved in such activities range from whales, to turtles, to seals, to sharks and rays, and interactions range from simply observing these animals from a boat or from shore to in water dive and snorkel experiences [10]–[15]. These activities have expanded, becoming increasingly popular since the 1980s [7], [10], [16], [17], and have been shown to generate significant economic benefits, both in their own right and to the supporting businesses within the local economies in which they operate [5], [18]–[20]. While management of wildlife-centred marine ecotourism presents its own challenges [21], well-managed models have proven to generate sustainable livelihoods, potentially providing a long-term solution for conserving marine megafauna [9], [22]. In some locations, marine ecotourism operations provide significant financial benefits to communities where few alternative sources of income exist [5], [23]. In many countries, manta ray interactions are proving to be a highly sought-after experience for divers and snorkelers [11], [24]–[26], with tourists in the Maldives willing to pay more for excursions involving mantas than either sharks or turtles [27], and the number of visitors on tours to see manta rays surpassing those looking for whale sharks in Western Australia’s Bateman Bay on Ningaloo Reef [25].

Manta rays belong to the family Mobulidae, a small, but diverse, family of planktivourous elasmobranchs (2 species within the genus *Manta* and 9 species within the genus *Mobula*, collectively referred to as “mobulids”) with a global distribution across tropical, subtropical and temperate waters [28]. The genus *Manta,* collectively known as manta rays, has recently been re-described and comprises two recognised species, the reef manta, *Manta alfredi,* and the giant manta, *Manta birostris,* with a third putative species, *Manta* cf *birostris,* believed to occur in the Caribbean Sea, Gulf of Mexico and the east coast of the United States [29]. *M. birostris* is the largest of all the mobulids reaching wingspans (referred to as disc width or DW) of over 7 m with the slightly smaller *M. alfredi* growing to around 5 m DW [29]. All 11 mobulid species are harmless to humans, feeding predominantly on zooplankton, with the *Manta* species in particular often aggregating predictably and seasonally to feed, visit cleaning stations or mate [28]. Their large size, predictable patterns of occurrence, and perceived friendly and curious nature combined with the relative safety of interacting with a harmless animal has resulted in the aforementioned popularity with divers. A detailed study of manta ray watching in the Maldives estimated tourist expenditures of US$8.1 m annually for manta ray dives and snorkels [11].

Unfortunately these same characteristics that attract divers and snorkelers (i.e. predictable nature, propensity for surface feeding, large size and lack of human avoidance), also make them a relatively easy target species for fishers in some parts of the world [28]. Both species of manta ray are considered unsustainable as a fishery resource due to several elements of their life history such as late maturity, long lives and exceptionally low fecundity (only one pup on average every two to three or more years [28], [30], [31] (G. Stevens, pers. comm.)). These characteristics not only make them vulnerable, but also considerably reduce their ability to recover population numbers when depleted [28], [30]–[34]. Populations in a number of countries’ waters could be vulnerable to local extinction [30], [31], [33]–[36], and certain monitored subpopulations, including Gulf of California, Mexico, Indonesia and the Philippines, have been rapidly depleted [37]–[39]. While the meat of these animals is deemed to be of poor quality and is worth little [11], [28], [35], [40], the gill plates or branchial filaments have become highly sought after in Asian markets [30], [31], [35]–[37] where they are utilised in a tonic marketed to treat a wide variety of conditions [35]. A 2011 report on this trade estimated the value of this market at US$11.3 m per year across all mobulid species [35], with an estimated US$5 m from *Manta* species alone (S. Heinrichs, pers. comm.). As further evidence of increased fishery pressure on these species, the FAO reported that mobulid catches increased from 900 to 3300 tonnes over the period 2000–2007 [41], with additional unreported catches likely [37]. While manta rays face other threats, including bycatch in non-target fisheries, boat strikes, entanglement and natural predation [28], it is targeted fisheries, which pose the greatest threat to their survival.

Although legal protection for manta rays has been limited and not always well enforced, recent measures adopted by two important international treaties governing the conservation and trade of threatened species, represent significant progress and indicate a greatly increased level of awareness of manta rays as species of international conservation concern. In response to a proposal by Ecuador, in November 2011, the giant manta was included on both Appendix I and II of the Convention on Migratory Species (CMS), a non-binding agreement of 116 governments to “strive towards strictly protecting” the species. In March 2013, a proposal sponsored by Ecuador, Brazil and Colombia to include the genus *Manta* on Appendix II of the Convention on International Trade in Endangered Species of Wild Fauna and Flora (CITES) was adopted by the 16^th^ Conference of the Parties. The 178 Parties to this binding treaty will now be required, following the implementation period, to demonstrate that any exports of manta rays or their parts have been obtained from legal and sustainable sources. Laws prohibiting the catch or trade of one or both *Manta* species have been passed in one region (the European Union), six countries (Ecuador, Mexico, Philippines, Maldives, New Zealand, and Australia), two US States (Hawaii and Florida), two US Territories (Guam and the Commonwealth of the Northern Mariana Islands), the state of Yap, Federated States of Micronesia (FSM), and a few small Marine Park Areas. These national and local measures only cover a small proportion of the ranges of these highly migratory species, however, leaving them vulnerable to a number of unregulated fisheries.

In light of the threats that exist to these animals, this study aims to provide the first global estimate of the direct economic impact of manta ray watching tourism and examine the potential socio-economic benefits of non-consumptive manta ray watching operations relative to consumptive use of manta rays as a fishery resource.

## Methods

### Study Definitions and Terms

The following definitions refer to the key terms used throughout this study. *Manta ray watching* refers to recreational activities undertaken to view manta rays in the wild, which for this study includes dives and snorkels at manta ray dive sites, but could also potentially include observing manta rays from a boat. *Manta ray watching locations* are identified as locations where commercial operations conduct dives and/or snorkels at dive sites where manta rays are a primary attraction, consistent with criteria used in other tourism valuation studies (e.g. [10], [11]). Locations and individual dive sites where divers encounter manta rays opportunistically were not considered as manta ray watching activities, and in keeping with the conservative approach taken by Anderson et al. [11], visits to manta ray sites during times of year when manta rays are not seen consistently were also excluded. *Direct economic impact of manta ray watching* comprises direct expenditures (we used gross expenditures as have most of the comparable economic valuation studies reviewed [5], [6], [11], [12], [14], [17], [19], [20], [42]–) to dive businesses from manta ray dives and snorkels (referred to hereafter collectively as *manta dives*) and associated tourism expenditures, which together provide a conservative estimate of total tourist expenditures on manta ray watching activities [17]. *Associated tourism expenditures* include the proportion of tourist expenses, such as lodging, food and other purchases that can be attributed to the manta dives [17], [19], [20], [47]. While we only considered in-country expenditures to estimate direct economic impact per country, considering the remote locations of most manta watching locations, it is likely that a large proportion of manta ray watching tourists travel long distances, and therefore international travel expenses not included in this analysis may contribute substantially to economic impact globally.

### Data Collection

From August 2011 to August 2012, data on the extent of manta ray watching and expenditures on manta dives were collected through primary and published research (Table 1) using a five step process; (1) literature review to identify existing published and unpublished estimates of manta dive expenditures, using Google Scholar, the Manta Trust group on Mendeley.com, and the resource pages of manta ray research organisations’ websites, (2) broad level Internet research to identify manta ray watching locations, conducted through review of manta ray research organisations’ websites and the IUCN Red List assessments for both *Manta* species to identify countries and locations where manta ray sightings have been documented, (3) location specific Internet research to identify dive operators and manta dive sites, conducted with the Google search engine using key word terms: “location+manta ray dives”, “location+dive sites+manta”, “location+dive operators”, “location+dive shops”, “location+live aboard diving”, “location+dive resorts”, (4) questionnaires emailed to dive operators (File S1) to collect information on manta dive expenditures and additional data, and (5) personal interviews with select operators and area experts to review and verify results for each location. Depending on the quality of information available via the different data collection methods in each destination, subsequent stages of investigation were adapted to allow comparable quality of data to be collected for all countries and locations. For example where dive operator websites provided very detailed information, the area specific Internet research was the primary source of data, with survey responses and personal interviews used for verification. In other locations, especially those with a small number of operators or where only limited information was available online, survey responses and personal interviews were the primary data sources.

**Table 1:** Data Collection: Details collected and sources.

Details	Internet research	Operator surveys	Other sources*
Manta watching tourism locations	x		x
Number of operators offering manta dives	x	x	x
Dive sites considered to be primarily manta dive sites	x	x	x
Seasons that manta rays are present (if seasonal)	x	x	x
Number of trips made to manta dive sites per year	x	x	x
Maximum number of divers per manta dive trip	x		
Average number of divers per manta dive trip		x	x
Average number of divers visiting manta sites		x	x
Price per manta dive	x	x	x
Number or proportion of dives/days lost due to poor weather or other factors		x	x
Operator perception questions		x	

* Other sources include manta ray researchers and dive travel booking agents.

For each manta ray watching location we focused on obtaining the following details; (1) number of operators offering manta dives, (2) dive sites considered to be primarily manta dive sites, (3) seasons that manta rays are present (if seasonal), (4) number of trips made to manta dive sites per year, (5) maximum number of divers per trip (capacity) and average occupancy rates, (6) price per dive, (7) the number or proportion of dives/days lost due to poor weather or other factors, and (8) operator perceptions with regard to the importance of manta rays to their business and the local community and how manta rays rank among sea life that divers most want to see. As an additional verification step, operators were also asked if they knew how many other dive operators visited the manta dive site(s) and if they were able to estimate the total number of dives to the site(s). Two survey versions were designed, one for day boat operations and one for live-aboard boats, and questions were often personalised to reflect any data already gathered or specific questions that arose through the Internet research. Most surveys were conducted in English, but for some areas were translated to the local language.

To estimate associated tourism expenditures, a benefits transfer approach was employed based on methods used by Hoyt [17] to estimate direct economic impact of whale watching in locations where detailed data on associated tourist expenditures were not available. Hoyt [17] applied ratios of “total expenditures” (whale watching tickets plus associated tourist expenditures) to “direct expenditures” (whale watching tickets) based on estimates of total expenditures from published whale watching studies for whale watching in comparable locations. Economic valuation literature supports use of the benefits transfer approach (or “value transfer”) in the absence of primary data (i.e. data collected directly from the study site), yet stresses the importance of ensuring that values are transferred across comparable sites or that adjustments are made to reflect differences in characteristics from the original study site to the site to which the values are being transferred [47], [48]. To ensure the values transferred would be comparable, we used expenditure data from studies focused on similar marine tourism activities, mainly shark diving or whale watching, and transferred values from the same countries (and regions within the country where available) or, in a few cases, to countries with similar tourism industry characteristics. Our study collected country specific data on dive tourist expenditures from one country tourism authority report [49], 10 published studies on the economic impact of tourist trips focused on viewing sharks [6], [10], [12], [18], [19], [42]–[44], [46], [50], and one study on the economic impact of whale watching tourism [20]. From each of these studies, we extracted average total expenditure per trip (and/or per day) and average expenditure on dives/whale watching tickets for each location analysed.

### Data Analysis

Depending on the amount and quality of data collected for each location, two methods were employed to estimate manta dive expenditures; Analysis 1- sum of the estimated annual manta dive expenditure values for each operator in the area [17], [20] and Analysis 2- estimate based on the total number of boats and divers visiting the manta dive site(s) from dive operator surveys and interviews, adapted from Anderson *et al.* [11]. The first calculation method was used for all areas for which we were able to collect sufficient data to calculate individual estimates for each operator. Analysis 1 used two formulas, one for day boat dive operations (manta dives per week × weeks per season × average price per dive × average number of guests per dive) and one for live-aboard dive operations (cost per trip/number of dives per trip = cost per dive; cost per dive × number of manta dives per trip × average number of divers per trip × number of trips per season). Analysis 2 used the following formula, and averaged the results when input from more than one operator was available: Number of boat visits to the manta dive site(s) per week (number of boats × visits per week) × Average number of guests per boat × Number of weeks in the season = Estimated manta ray dive revenue per season. For both methods any time lost due to poor weather conditions or other factors was factored in to ensure that expenditures were not over estimated.

Analysis of raw data and/or estimates of manta dive expenditures provided by local researchers employed the same or more precise methods. The Yap estimate was based on actual data from the largest operator plus his estimate for the other operators (B. Acker, pers. comm.), and the data source for the W. Australia estimate was government figures for the actual number of dives at the manta ray site multiplied by the average cost per dive trip, which was relatively constant across operators (F. McGregor, pers. comm.). Researchers in Kona, Hawaii collected actual manta dive expenditure data through a survey process, involving a phone call or personal visit to every manta ray dive operator in the area (J. McLaughlin, pers. comm.), and the Japan revenue figure was obtained by summing estimates provided by a local expert of the number of boats, dives and snorkels visiting each of the manta ray dive sites for every day of the year. The expert providing these estimates (T. Ito) has been diving these sites almost daily for the past 30 years (T. Kashiwagi, pers. comm.).

To calculate estimates of direct economic impact including associated tourism expenditures, we determined ratios from the reviewed literature that would best represent average local expenditures by manta ray watching tourists compared with their average expenditures on manta dives. We applied these ratios to the manta dive expenditure figures to estimate the direct economic impact of manta ray watching for each country. For example, Vianna *et al*. [50] provided details on average dive tourist expenditures for diving focused trips in Palau of $2,081 per trip, and expenditure on dives of $749 per trip, resulting in a ratio of 2.78∶1, that is $2.78 being spent across all trip expenditures for every dollar spent directly on diving. Applying this ratio to the expenditures on manta dives in Palau from our study, we were able to estimate the direct economic impact of manta ray watching for Palau. Since data on diver expenditures were not available for every manta ray watching location, we calculated ratios for most countries using total daily expenditure for whale watchers from O’Connor et al. [20] and modified these figures to account for the higher costs of manta dive trips compared with whale watching trips. For example, in Mozambique, the average daily cost of whale watching per participant was $58 for the whale watching ticket plus $85 in associated expenditures for a total direct economic impact of $143 per participant per whale watching day, a ratio of 2.46∶1. Modifying these figures to account for the higher cost of manta dives, the total direct economic impact comes to $203 ($118 cost of manta dives+$85 associated expenditures) per manta ray watching day and a ratio of 1.71∶1 ($203/$118).

In order to verify that “our methods were robust and our estimates plausible as compared with other similar studies” [51], we reviewed 26 published studies and reports estimating the economic value of marine based tourism activities, most of which focused on marine megafauna viewing operations [5]–[7], [10]–[12], [14], [17]–[20], [42]–[47], [49]–[57], and checked results by comparing estimates and important data points obtained from multiple sources. As an additional verification step, we compared estimates obtained from both methods for 4 locations (Palau; Madagascar; Bora Bora, Fr. Polynesia; Yap, FSM) and checked data points from our estimates against official figures where available (Palau [5], [50], [58]; Raja Ampat, Indonesia [59]; Similan-Surin Islands, Thailand [60]; FSM [61]). Finally, all values have been converted into US$ for consistency using current exchange rates from www.xe.com.

## Results

From the literature review, we identified a single peer-reviewed published estimate on manta ray watching in the Maldives [11]. Manta researchers and local experts provided estimates of manta ray watching dives and expenditures or sufficient details to enable us to calculate estimates for Bateman Bay, Western Australia (F. McGregor), Yap, FSM (B. Acker), Yaeyama Islands, Japan (T. Kashiwagi, T. Ito) and Kona, Hawaii, United States (Manta Pacific Research Foundation). Data and estimates for all other locations were obtained from Internet research and dive operator surveys.

### Extent of Manta Ray Watching Tourism

This study identified and investigated operations in 31 countries where manta dives were found to take place. Of these countries, 25 met our criteria to be eligible for estimation of manta dive expenditures, and of these, we were able to make or locate estimates for 23. In five of the six countries excluded from analysis (Egypt, South Africa, Sri Lanka, New Zealand and Tonga), despite the fact that manta rays were seasonally encountered and were considered to be an important motivation for a proportion of clients selecting these locations, no primarily manta dive sites had been established and manta rays were not seen with enough regularity to enable dive operations to market manta ray specific dives. In the case of Sri Lanka, the political history of the country has meant that dive tourism is at present only an emerging industry, and while no cleaning stations or other sites where manta rays can be encountered reliably have yet been identified, efforts to locate such sites were reported to be underway. In the Cook Islands, the area where manta rays aggregate, Suwarro Bay, is only accessible currently by private yacht and no commercial dive tourism has been established to date. In Tanzania, Internet research confirms manta ray watching tourism, but we were unable to collect sufficient data to make an expenditure estimate. In two emerging manta ray watching locations, Laje de Santos in Brazil and Isla de la Plata in Ecuador, socio-economic studies are underway by local researchers, but expenditure estimates are not yet available.

**Figure 1:**
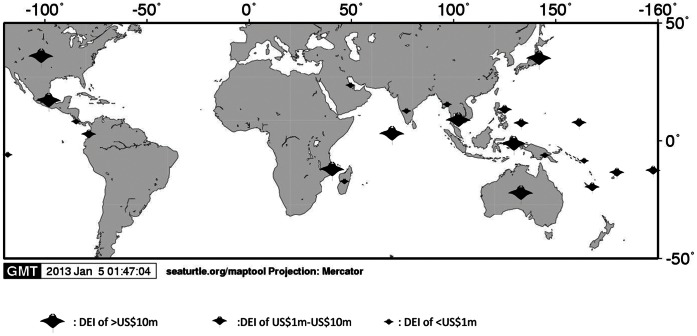
Global distribution and direct economic impact (DEI) of manta watching tourism. Direct economic impact comprises estimated tourist expenditures on manta ray dives and associated expenditures, such as lodging, food and local transportation, which can be attributed to manta ray diving.

This study showed manta ray watching tourism to be widely distributed, present across 6 continents and numerous island nations, specifically occurring at approximately 200 different identified manta dive sites (Figure 1). Of the 23 countries examined, we estimated over one million manta ray dives and snorkels per year (Table 2, Table S1). Through our Internet research and surveys, we identified 386 operators that take divers and snorkelers to manta sites in these countries, studied the websites of 319 of these operators (82.6%), and sent surveys to 244 (63.2%). Ninety-four of the surveys were completed and returned (39% response rate). In addition to providing data on their operations, the dive operators who responded were frequently very helpful with reporting additional operators we had not located through web research and filling in gaps in data.

**Table 2:** Manta Ray Watching Tourism Extent and Dive Expenditure Estimates (US$).

Country	Number of MantaDive Sites	Estimated Total Annual Manta Dives	Estimated Total Annual Expenditure (US$)	Sources
Japan	3	145,158	$11,400,103	T. Kashawagi, pers. comm.
Indonesia	11	139,594	$10,655,022	Internet and Surveys
Maldives	91	157,000	$8,100,000	Anderson *et al.*, 2011
Mozambique	18	129,102	$7,640,351	Internet and Surveys
Thailand	3	121,767	$7,418,750	Internet and Surveys
Australia	14	75,393	$6,529,435	Internet, Surveys & F.McGregor, pers. comm.
Mexico	4	40,680	$5,084,600	Internet and Surveys
United States	3	50,912	$4,661,938	Manta Pacific Research Foundation, pers. comm.
Federated States of Micronesia	6	67,872	$4,091,520	B. Acker, pers. comm.
Palau	2	35,390	$2,455,108	Internet and Surveys
French Polynesia	3	17,550	$1,367,625	Internet and Surveys
Philippines	8	18,463	$863,479	Internet and Surveys
Ecuador*	1	2,557	$726,126	Internet and Surveys
Fiji	5	14,967	$630,148	Internet and Surveys
New Caledonia	2	5,100	$524,988	Internet and Surveys
Solomon Islands	3	2,908	$319,332	Internet and Surveys
Madagascar	1	5,426	$206,498	Internet and Surveys
India	2	979	$198,890	Internet and Surveys
Papua New Guinea	2	2,012	$175,561	Internet and Surveys
Myanmar	3	2,158	$157,606	Internet and Surveys
Costa Rica	2	2,184	$109,200	Internet and Surveys
Kiribati	2	350	$17,500	Internet and Surveys
Sudan	1	181	$13,506	Internet and Surveys
TOTAL	190	1,037,703	$73,347,286	

* Ecuador has a second manta dive site in Isla de la Plata, but dive expenditures for this site are not included in the Ecuador estimate.

### Direct Expenditures on Manta Dives

This study estimated direct expenditures on manta dives in the 23 countries analysed at over US$73 million annually, with ten countries accounting for almost 93% of the global expenditure estimate, specifically Japan, Indonesia, Maldives, Mozambique, Thailand, Australia, Mexico, United States, Federated States of Micronesia and Palau (Table 2, Table S1). Estimates from Japan alone accounted for over 15% of the total estimate with manta dive expenditures in this country estimated at $11.4 million generated from 3 dive locations. In Indonesia expenditures of over $10.6 million were estimated from 11 sites in four key locations, and in contrast Anderson *et al.* [11] estimated expenditures of $8.1 million from 91 sites in 12 atolls in the Maldives.

### Direct Economic Impact of Manta Ray Tourism

Direct economic impact of manta ray watching tourism was estimated at $140 million annually (Table 3, Table S2). Analysis of the expenditure data from the dive studies and country report from 6 countries resulted ratios ranging from 1.67× to 3.43× and a median value of 2.00× to 2.46×. These sources provided trip expenditure data for 7 of the 23 manta ray watching countries (Australia, Fiji, French Polynesia, Palau and Thailand, including Myanmar and India, as manta dive trips to these countries originate in Thailand with Thai operators), values from O’Connor et al. [20] were used to calculate ratios for an additional 13 countries (Costa Rica, Ecuador, FSM, Indonesia, Japan, Madagascar, Maldives, Mexico, Mozambique, New Caledonia, Philippines, Sudan (based on Egypt as these trips originate in Egypt with Egyptian operators) and United States), and we applied ratios from countries with similar tourism characteristics for the remaining 3 countries (Kiribati, Papua New Guinea, Solomon Islands). The ratios calculated from the whale watching studies (and adjusted for manta dive costs) ranged from 1.42× to 3.31× with a median value of 1.84× to 1.91×, indicating that associated expenditures related to diving and whale watching appear to be comparable.

**Table 3:** Direct Economic Impact of Manta Ray Watching Tourism.

Country	Manta Dive ExpenditureEstimate (US$)	Trip Expenditure : Dive Expenditure	Direct Economic Impact Estimate
Japan	$11,400,103	1.56	$17,784,161
Indonesia	$10,655,022	1.42	$15,130,131
Maldives	$8,100,000	1.91	$15,471,000
Mozambique	$7,640,351	1.71	$13,065,000
Thailand	$7,418,750	1.67	$12,389,313
Australia	$6,529,435	2.23	$14,560,640
Mexico	$5,084,600	2.01	$10,220,046
USA	$4,661,938	3.31	$15,431,015
FSM	$4,091,520	1.92	$7,855,718
Palau	$2,455,108	2.78	$6,825,200
Fr. Polynesia	$1,367,625	2.71	$3,706,264
Philippines	$863,479	1.64	$1,416,106
Ecuador	$726,126	2.56	$2,009,411
Fiji	$630,148	2.52	$1,587,973
New Caledonia	$524,988	2.21	$1,160,223
Solomon Islands	$319,332	1.92	$613,117
Madagascar	$206,498	1.84	$379,956
India	$198,890	1.67	$332,146
Papua New Guinea	$175,561	1.92	$337,077
Myanmar	$157,606	1.67	$263,202
Costa Rica	$109,200	2.50	$273,000
Kiribati	$17,500	1.92	$33,600
Sudan	$13,506	1.69	$22,825
Totals	$73,347,286		$140,716,597

### Verification of Results

Our verification measures showed that our results were comparable across the techniques we used and to the official data available. In Palau, for example, we compared manta ray dive expenditure results from summing individual operator estimates (Analysis 1) to two calculations from estimates of total number of boats and divers provided by a local researcher and one of the dive operators (Analysis 2). These three estimates were comparable with only slight variations (Researcher: $2.44 m; Dive Operator: $2.71 m; Surveys: $2.45 m). In Yap, one operator completed our dive survey in addition to supplying his own estimate for all of Yap, with the two resulting estimates differing by only 1%. Where available, we also checked data and estimates against official sources. For Raja Ampat, we compared the number of divers from Raja Ampat 2011 entrance fee records (7,667) [59] to our estimate of the total number of divers (6,472), and found only an 18% variance in the two numbers. For Palau, we also compared this study’s estimated number of divers to published data [5], [50], [58]. In this case, our calculation yielded an estimate of 39,280 divers as compared to the average number of divers from 2007–9 of 40,976 from Vianna et al. [5], [50] and 55,619 divers in 2011 from Palau Visitors Bureau data (assuming 51% of total visitors are divers per Vianna et al. [5], [50]), indicating that both of these estimates were conservative.

### Dive Operator Survey Responses

Operator responses (N = 94) gave valuable insights into the perceived value of manta rays to their businesses and local communities and the motivation of tourists who chose to participate in these activities (Table 4). All dive operators responding to our survey ranked manta rays as one of the top five attractions for divers with 87% (N = 82) ranking them in the top 3 and 29% (N = 27) ranking manta rays as the number one attraction for divers. One international live aboard dive company with operations in top dive destinations throughout Asia and the Pacific reported that across all locations customer requests for manta rays were second only to whale sharks (S. Erbe, pers. comm.). All of the operators surveyed perceived manta rays to be important to their business and to the local community, and many added that customers frequently ask about manta rays and consider the opportunity to see them as important in their decision to book a dive trip. One operator from Raja Ampat, Indonesia reported that telling potential guests about the nearby manta ray cleaning station is a “clincher” to the guest confirming a trip (M. Miners, pers. comm.). In Indonesia, because manta rays are often sighted during low tourism season, operators in Bali and Komodo described manta rays as “vital to keeping the business going” during otherwise slow times (L. Harding pers. comm., B. Pilkington-Vincett pers. comm.). Fishermen and local hotels on Nusa Lembongan (Bali) have also been able to supplement their income by taking snorkelers to see manta rays (C. Guillevic, pers. comm.). A dive operator, who runs trips to the Revillagigedos Islands in Mexico, stated that while 100% of the dives on these trips are not “manta dives”, without manta rays, they would have no business at this destination (M. Lever, pers. comm.).

**Table 4:** Responses to Survey Questions on Dive Operator Perception Questions.

	Number of Responses	Percentage of Respondents
1. Importance of manta rays to business and local communities (n = 84)		
Yes	84	100%
No	0	0%
2. Manta rays’ rank among sea life that divers most want to see (n = 94)		
Top 1	27	28.7%
Top 3	55	58.5%
Top 5	12	12.8%
Total respondents ranking mantas in top 5	94	100%

Of the 94 responses received, 70 (74.5%) provided additional comments in response to the following general request: *“Please provide any other comments you’d like to add that might be helpful with assessing the value of manta rays to your business and to the local community overall”*. Fifty comments (71.4%) reiterated the importance of mantas to operators’ businesses and the high level of interest in manta encounters expressed by clients, and reported participation in local conservation efforts. Sixteen of the comments (22.9%) reported manta fishing (legal and illegal) in the vicinity of manta dive sites, perceived declines in manta sightings, and perceived negative impact to their business from decreased manta encounters. Specifically, operators from Indonesia (3) and the Philippines (2) reported seeing manta rays in local fish markets, while operators from Mozambique (3) reported targeting of manta rays near manta sites (even “on the house reef”), and operators from Thailand (1) and Mexico (2) reported witnessing illegal fishing of manta rays near manta dive sites in Mu Koh Similan National Marine Park in Thailand and in two locations in Mexico, where manta rays are legally protected. The one dive operator in Kiribati reported devastating declines in the local manta ray population as a result of bycatch from gill nets targeting other species placed in a channel frequented by mantas. Finally, four comments (5.7%) expressed concern about possible negative impacts of overcrowding at dive sites and some operators’ lack of compliance with established guidelines. Because these comments were not submitted in response to standard questions, they do not provide a scientific measure, but do represent issues the operators perceived to be important to assessing the value of manta rays to their business and the local community.

## Discussion

### Direct Economic Impact of Manta Ray Watching Tourism

Direct economic impact estimates from this study confirm that manta ray watching tourism generates significant economic benefits in the countries where it is based. These estimates are likely to be conservative in that they only take into account the number of manta dives as a percentage of total dives per dive trip and dive operator surveys confirmed that manta rays were an important factor in participants’ decision making and the only reason or the overriding reason in several top manta locations (Revillagigedos Islands, Mexico; Christmas Island, Kiribati; Yap and Pohnpei, Federated States of Micronesia; and Sangalaki, Indonesia).

Our research also suggests that manta ray watching provides additional economic benefits, which are not as easily quantified. For example, indirect economic impacts, “economic multipliers”, are the ripple effects tourism businesses generate throughout a local economy from purchasing goods and services and employing people, who in turn spend their wages to purchase goods and services in the community [14], [47], [62]. In Gansbaai, a popular shark viewing destination in South Africa, local retailers and other service businesses reported that tourists account for approximately 50% of their annual sales, thus providing significant economic benefits and jobs to businesses ranging from petrol stations to grocery stores to agricultural production [46]. Stoeckl et al. [51] notes that direct economic impact estimates for small regional economies can be viewed as estimates of total economic impact that are biased downwards by up to 25%. The marketing value of manta rays may also be considerable, as evidenced by the manta ray photos featured prominently on operator websites in all of the manta ray watching tourism areas included in this study. Even many areas that were excluded due to inconsistent manta ray sightings advertised the possibility of manta ray encounters, demonstrating the perceived value to dive businesses of even limited opportunities for manta ray encounters. In addition, the future growth potential for this industry could be substantial, as some of the most popular sites for viewing marine megafauna have only been discovered in recent years [56], [63], and our research identified locations in India, Sri Lanka, Indonesia, the Cook Islands, Marshall Islands and Tonga where manta rays are encountered, but tourism operations and infrastructure are still very limited or manta dives sites have not yet been identified.

### Potential Negative Impacts from Manta Ray Watching Tourism

Four of the operators surveyed reported concern about overcrowding at some manta sites, fearing possible negative impacts to the manta rays’ behaviour, and one also noted that manta ray sightings had decreased at very crowded sites. While some studies on marine megafauna tourism have suggested that improperly managed tourism might have negative implications for these animals [11], [21], [64], the results from studies that have attempted to quantify the effects of disturbance due to human interactions, have not yielded conclusive results [21], [64], [65]. In addition, much discussion surrounds the understanding of the terms involved in qualifying disruptive behaviour or harassment of animals, with people imposing their own values when interpreting such terms [66]. Further, in light of the substantial economic and conservation benefits attributed to marine megafauna ecotourism [10], [20], [52], [67], Hammerschlag et al. [68] recommend that sensitivities to disturbance be examined on a species by species basis in order to develop best practices for ecotourism most applicable to each species. In the Maldives studies have been carried out specifically to gain a better understanding of human-manta interactions and reactions, and to date disturbance to these animals appears to be minimal (Manta Trust, unpublished data). The findings from these studies will be used to inform a scientifically sound code of conduct for these species (G. Stevens, pers. comm.). Such guidelines, combined with educational and interpretive briefings, have been demonstrated to minimize tourists’ impacts on the environment and marine life while also enhancing their enjoyment of the experience [21], [69], and ‘user pays’ policies can be employed to cover the costs of these programs [9]. Deployment of such models in all manta ray tourism locations could ensure the welfare of the animals as well as continued customer satisfaction and business success.

### Direct Economic Impact of Manta Tourism Relative to Fisheries and Trade

It is increasingly evident that large charismatic marine animals are more valuable as long-term sources of tourism revenue than as onetime revenues to fisheries [5], [6], [10], [11], [26], [33], [45], [67]. For manta rays this study’s global estimate of direct economic impact of $140 million per year from tourism greatly exceeds the trade in manta ray gill plates estimated at $5 million per year (S. Heinrichs, pers. comm.; total trade in mobulid gill plates estimated at US$11 million [35]). Indonesia ranked as one of the top 3 destinations in the world for manta ray watching, with estimates of manta dive expenditures close to US$10.7 million and direct economic impact over US$15 million per year. Yet fishery surveys conducted in Indonesia over the past ten years provide evidence of unsustainable mobulid fisheries and associated population declines [35]–[37] (Setiasih et al. unpublished data), which may threaten these valuable manta ray watching tourism businesses. Based on analysis of landings data collected through surveys of ports in Lombok, Pelabuhanratu, Cilacap, Kedonganan, Lombok and Lamakera [35], [37] (Setiasih et al. unpublished data), the total annual income from manta ray fisheries in Indonesia is estimated at approximately US$442,000 (estimated 94% from gill plates for export; 6% from other products sold locally), less than 3% of the annual expenditures on manta ray watching tourism (M. O’Malley unpublished data). Additionally the tourism revenue figures estimated in this study only account for existing manta ray watching operations, without considering potential for development of these activities in other parts of the country. These figures also do not account for consumer surplus, though dive operator surveys in this study suggest that consumer surplus clearly exists for manta rays as an input to a recreational experience. Overlooking this important aspect of economic value may greatly understate the comparative benefit of non-consumptive use, since competitive conditions likely eliminate any producer surplus in consumptive use (D. Letson, pers. comm.).

As another means of demonstrating the disparity between the revenue iconic species can generate for tourism relative to consumptive use, published studies have used various methods to calculate the estimated lifetime tourism value per animal [5], [6], [11], [26], [63], [64]. However, these comparisons have been criticized as being simplistic [11], and might potentially be construed as weakening rather than strengthening the case for conservation, since a smaller number of animals yields a higher per animal value (70). Nevertheless per animal estimates can provide an effective means of communicating the worth of these animals to tourism compared with a fishery, which can be easily understood by policy makers and the general public [6]. Norman and Catlin [63] estimated the lifetime tourism value per whale shark in Australia at US$282,000, while the average landed price for a whale shark in Taiwan has been reported as US$3,500 [71]. This estimate was derived by dividing the estimated value of the whale shark tourism industry in Ningaloo Reef, Western Australia, the longest established whale shark watching destination, by the number of whale sharks associated with the site, and then multiplying by the generation time for this species, which approximates the number of years it takes for an animal to replace itself in the population.

Applying this method to manta rays in Yap, which is likely to be the longest established manta ray watching tourism destination, based around a stable population of approximately 100 reef manta rays [72], and the estimated generation time for manta rays of 25 years [30], [31], the lifetime value of each manta in Yap can be estimated at approximately US$1.9 million ($7.68 m/100×25). Anderson et al. [11] used a more conservative method to estimate individual values for a core group of manta rays that frequent a popular manta dive site in the Maldives at US$100,000 per animal over a 20-year period. Despite the varied results obtained from different calculation methods and different locations, both demonstrate the stark contrast to the average price for a whole manta ray (2.5 m DW) sold in a fish market in Sri Lanka, which is reported as US$41 [40], or the approximately US$200 a fishing crew in Lamakera, Indonesia receives for a large manta ray (5 m DW) [35].

### Potential Socio-economic Benefits of Manta Ray Tourism Relative to Manta Ray Fisheries

While the economic impact argument in favour of manta ray tourism relative to manta ray fisheries is clear, the socio-economic realities involved with communities shifting from manta ray fishing to manta ray tourism are more complex. However, potential negative impacts of manta fisheries on sustainable and more valuable manta tourism businesses that are important to other communities, and recent international agreements to protect manta rays and require strict regulation of trade, highlight the importance of investigating alternative sources of income for communities that derive a portion of their income from manta fisheries and trade. As fishing is a traditional way of life in many coastal communities, it’s important to note that presenting ecotourism as an alternative to unsustainable fisheries does not suggest that tourism replace all fisheries. Ecotourism can be compatible with these traditions and even help to support sustainable fisheries, as Vianna et al. [5] demonstrated that local fishers could earn more from supplying fish to feed shark diving tourists than from fishing for sharks. However, fishing that targets the iconic species tourists are coming to see and experience, is not compatible with ecotourism. For example, Orams [14] found that substantial tourism revenues from whale watching in Tonga would be severely impacted if whaling activities were resumed there.

In locations where mantas can be encountered reliably, ecotourism may provide one alternative to help communities shift towards more sustainable sources of income. In Lamakera, a small village in the Nusa Tenggara Timur Province, Indonesia, research suggests that a seasonal fishery targeting manta and mobula rays for the gill plate export trade may be the world’s largest manta ray landing site [35], and contributes significantly to the annual income of a small number of fishers (Setiasih et al. unpublished data). Located along an important pelagic corridor, seasonal migrations of manta and mobula rays and other megafauna highly valued in marine tourism, including whale sharks and several cetacean species [73], [74], [75], could potentially support the development of ecotourism in this location (Setiasih et al. unpublished data). While infrastructure investment would be required and there would be cultural challenges to overcome, tourism development in cooperation with fishing communities has been successful in Indonesia and other countries, in areas where manta rays and sharks are now important tourist attractions [22] (A. and M. Miners, pers. comm). In West Papua Province, Indonesia, manta rays and sharks are among the top tourist attractions in a 1220 square kilometre conservation zone, which was established through lease agreements between villages, who own the fishing rights for the area, and a dive eco-resort, which is built on an island previously used as a shark finning camp. Locally-hired rangers, some of whom were formerly engaged in shark finning and other unsustainable fishing practices, enforce the conservation zone, and the villages benefit from lease fees, employment (supporting 80 families from the local villages), the resort’s purchases of local produce and fish, community projects and skills training programs, and improved fishing in the waters surrounding the no-take areas of the conservation zone (A. and M. Miners, pers. comm.). Recognition of the value and potential of marine ecotourism and the importance of marine megafauna to this industry has since led to adoption of legal protection at the Raja Ampat Regency level for manta and mobula rays, sharks, turtles and dugongs. Such community level agreements, in which dive operators and/or non-governmental organisations pay communities to restrict fishing in designated areas or adhere to agreed upon sustainable guidelines, can protect valuable species and habitats, while ensuring broader distribution of tourism benefits throughout the community, including to those not directly employed by dive resorts [22]. In the Maldives, even without such agreements, many fishermen shifted from shark fishing to more sustainable employment in dive tourism, as evidenced by 19 shark fishing boats on the Island of Dhangethi in south Ari Atoll in July 1991 and 22 boats involved in tourism and only 4 involved in shark fishing by August 1998 [45].

While development of manta tourism may not be a feasible alternative to manta fishing in all areas, measures to curtail these fisheries should nevertheless be pursued and other economic alternatives investigated. Most manta fisheries are reported to be somewhat opportunistic and not primary sources of income to the fishermen involved [40] (K. Forsberg, pers. comm., S. Heinrichs, pers. comm.), but offers from foreign traders of relatively large amounts of money for mobulid gill plates, especially those from large manta rays, have provided incentive for fishers to supplement their regular income by targeting manta rays they encounter during fishing trips [40] (S. Heinrichs, pers. comm.). However, this income is not likely to be sustainable in the long term, due to manta rays’ low reproductive capacity, and these fisheries will soon be required to demonstrate that they are sustainable and legal to comply with new export requirements, which may not be possible. In addition, Indonesian fishermen have reported targeting mobulids in the range of manta tourism areas in Indonesia and Australia (Setiasih et al. unpublished data), and dive operator survey responses and published reports suggest negative impacts to manta tourism operations where manta fishing activities overlap or are within the migratory range of the mantas on which the tourism operations depend. Operators reported targeted fishing and bycatch of manta rays in the vicinity of manta dive sites and significantly decreased manta ray sightings in locations in Indonesia, Thailand, the Philippines, Mozambique and Kiribati. Manta researcher observations and published studies also suggest large declines in diver sightings of one or both manta species in areas that overlap with manta fishing activities in Australia (F. McGregor, pers. comm.), Mozambique [76] and the Philippines [77].

### Potential Sources of Error

Due to the global scope of this study and resource limitations precluding extensive onsite surveys and data collection in all of the 23 manta watching countries identified, the figures presented in this study should be considered as estimates. As the first study to attempt such a global estimate of the direct economic impact of manta ray watching tourism, however, these estimates are still valuable to demonstrate the economic importance and potential of this activity and the benefits of conserving manta rays, even if only for economic reasons. We recommend further in-depth local socio-economic studies of manta ray tourism, which would enable more accurate estimates of economic impacts, including direct assessment of manta dive participants’ motivations for visiting the locations and their willingness to pay for the opportunity to see manta rays, to enable researchers to quantify estimates of consumer surplus. In locations with manta fisheries, local studies could also better assess the feasibility of manta tourism as an alternative to manta fisheries, and investigate other sustainable alternatives at the community level.

### Conclusions

The slow reproductive rate of manta rays and evidence of large declines associated with directed manta ray fisheries strongly suggest that revenues from catch and trade are likely to diminish and disappear over time. On the other hand, the demand for ecotourism focused on marine megafauna is reported to be growing [10], [20], [52], and the global tourism industry overall forecasts significant growth over the next twenty years [78]. Tourism is not expected to solve all the complex issues associated with unsustainable manta ray fisheries and the international trade in these threatened species, yet development of well managed manta ray watching tourism may offer a promising alternative in some of the areas where manta rays are still targeted.

While tourism operations must be managed properly and make efforts to reduce their ecological footprint, fisheries must do the same by not targeting species that cannot be fished sustainably, and taking sensible management measures to ensure that the species they do target are not depleted. Coastal communities depend heavily upon their surrounding marine resources and it is crucial that they strive to manage these resources wisely for themselves and future generations. For those communities in areas, which are frequented by manta rays, manta ray watching tourism can be an important aspect of their marine resource management plan that, if properly managed, can potentially provide sustainable benefits for many generations.

## Supporting Information

Table S1 **Manta Ray Watching Tourism Extent and Dive Expenditure Estimates.** Research results used to calculate the country totals summarized in Table 2.
(PDF)

Table S2 **Direct Economic Impact of Manta Ray Watching Tourism.** Research results used to calculate ratios of total expenditures to dive only expenditures to produce the country direct economic impact estimates summarized in Table 3.
(PDF)

File S1 **Dive Operator Surveys.** Survey questions sent to live aboard dive operators and land-based dive operators.
(PDF)
