# Supplementary material for: The Global Economic Impact of Manta Ray Watching Tourism
Source: PLoS One. 2013 May 31;8(5):e65051. doi: 10.1371/journal.pone.0065051 (PMC3669133; doi:10.1371/journal.pone.0065051)
Supplement: Table S1 — Manta Ray Watching Tourism Extent and Dive Expenditure Estimates. Research results used to calculate the country totals summarized in Table 2. (PDF) [file pone.0065051.s001.pdf]

Table S1. Manta Ray Watching Tourism Extent and Dive Expenditure Estimates (US$)

Country	Region	Data Source	Operator/ Trip type	Number of Manta Dive Trips per Season	Average Cost/ Dive	Average Number of Passengers	Estimated Number of Manta Dives	Estimated Manta Dive Revenue
				Trips to Manta Dive Sites x # Manta Dives per Trip	Value from Research	Value from Research	Number of trips x Avg Number of Passengers	Number of Manta Dives x Avg Cost/ Dive
Australia	Western Australia	Pers. Comm. Frazer McGregor (1 dive site)	NA	NA	$164	NA	21,306	$3,491,564
	Great Barrier Reef	Internet; Surveys; Researcher assistance (Lady Elliot Island - 1 operator, 5 dive sites)	Op. 1 - Dives	1,440	$64	12	17,280	$1,099,008
			Op. 1 - Snorkels	1,440	$42	16	23,040	$976,896
		Internet; Surveys; Researcher assistance (N. Stradbroke Island - 3 operators, 1 dive site)	Op. 1 - Dives - High Season	256	$64	11	2,816	$180,590
			Op. 1 - Dives - Low Season	32	$64	6	192	$12,313
			Op. 2 - Dives	64	$87	15	960	$83,952
			Op. 3 - Dives	8	$87	10	80	$6,996
		Internet; Researcher assistance (Heron Island - 1 operator, 4 dive sites)	Op. 1 - Dives - High Season	43	$72	43	1,849	$133,276
			Op. 1. - Snorkels - High Season	30	$51	43	1,290	$65,635
			Op. 1. - Dives - Low Season	108	$72	30	3,240	$233,539
			Op. 1 - Snorkels - Low Season	54	$51	30	1,620	$82,426
		Internet; Surveys (Lady Musgrave Island - 1 operator, 1 dive site)	Op. 1 - Dives	60	$74	10	600	$44,520

Manta Ray Watching Tourism Extent and Dive Expenditure Estimates (US$)

	Cocos-Keeling	Internet; Surveys (Christmas Island - 1 operator, 2 dive sites)	Op. 1 - Dives	280	$106	4	1,120	$118,720
**Australia Subtotal**							**75,393**	**$6,529,435**
Costa Rica	Catalina-Bat Islands	Internet; Surveys (3 operators, 2 dive sites)	Op. 1 - Dives	52	$50	7	728	$36,400
			Op. 2 - Dives	52	$50	7	728	$36,400
			Op. 3 - Dives	52	$50	7	728	$36,400
**Costa Rica Subtotal**							**2,184**	**$109,200**
Ecuador	Galapagos Islands	Internet; Surveys (4 operators, 1 dive site)	Op. 1 - Dives	42	$331	11	470	$155,673
			Op. 2 - Dives	8	$331	11	90	$29,652
			Op. 3 - Dives	78	$292	13	998	$291,200
			Op. 4 - Dives	78	$250	13	998	$249,601
**Ecuador Subtotal**							**2,557**	**$726,126**
Federated States of Micronesia (FSM)	Yap	Personal Communication Bill Acker; Verfied with Internet; Surveys (6 operators, 5 dive sites)	All ops. - dives	NA	$60	NA	66,000	$4,000,000
	Pohnpei	Internet; Surveys (2 operators, 1 dive site)	Op. 1 - Dives	208	$48	4	832	$39,520
			Op. 2 - Dives	104	$50	10	1,040	$52,000
**Federated States of Micronesia (FSM) Subtotal**							**67,872**	**$4,091,520**
	Taveuni	Internet; Surveys (2 operators, 1 dive site)	Op. 1 - Dives	52	$28	5	260	$7,326
			Op. 2 - Dives	52	$28	5	260	$7,326
	Savu Sea Koro Bay /	Internet; Surveys (5 [illegible]	Op. 1 - Dives	16	$25	10	160	$3,967
			Op. 2 - Dives	16	$25	10	160	$3,967
			Op. 3 - Dives	73	$31	4	292	$9,050

Manta Ray Watching Tourism Extent and Dive Expenditure Estimates (US$)

Fiji	Vita Livu	operators, 1 dive site)	Op. 4 - Dives	198	$66	9	3,366	$222,882
			Op. 5 - Dives	48	$59	17	1,613	$95,273
	Yasawa Islands	Internet; Surveys (3 operators, 1 dive site)	Op. 1 - Snorkels	20	$28	10	200	$5,635
			Op. 2 - Snorkels	80	$28	20	1,600	$45,083
			Op. 3 - Snorkels	20	$28	10	200	$5,635
	Kadavu	Internet; Surveys (4 operators, 2 dive sites)	Op. 1 - Dives	416	$31	8	3,328	$103,150
			Op. 2 - Dives	252	$36	4	1,008	$36,355
			Op. 3 - Dives	252	$34	4	1,008	$33,799
			Op. 4 - Dives	252	$34	6	1,512	$50,697
**Fiji Subtotal**							**14,967**	**$630,148**
French Polynesia	Bora Bora	Internet; Surveys (3 dive operators, 1 dive site)	Op. 1 - Dives	325	$101	13	4,117	$414,375
			Op. 2 - Dives	325	$101	7	2,422	$243,750
			Op. 3 - Dives	175	$94	2	391	$36,750
			Snorkels (all boats)	325	$67	7	2,422	$162,500
	Marquesas	Internet; Surveys (1 operator, 1 dive site)	Op. 1 - Dives	200	$87	2	373	$32,500
	Tikehau	Internet; Surveys (2 operators + 5 snorkel boats, 1 dive site)	Op. 1 - Dives	300	$84	4	1,118	$93,750
			Op. 2 - Dives	300	$82	4	1,118	$91,500
			Snorkels (5 boats)	300	$52	19	5,589	$292,500
**French Polynesia Subtotal**							**17,550**	**$1,367,625**
India	Andaman Islands	Internet; Surveys (2 operators, 2 dive sites); 1 add'l land-based operator makes trips very occasionally, not inc.	Op. 1 - Dives	48	$195	14	685	$133,791
			Op. 2 - Dives	16	$222	18	294	$65,099
**India Subtotal**							**979**	**$198,890**
			N. Lembongan (10) - Dives - High Season	1,370	$51	8	11,363	$574,091
			N. Lembongan (10) - Dives - Low Season	1,700	$51	6	10,575	$534,282

Manta Ray Watching Tourism Extent and Dive Expenditure Estimates (US$)

	Bali	Internet; Surveys (55 operators, 1 dive site)	N. Lembongan (10) - Snorkels	3,000	$21	5	15,552	$329,249
			Sanur, Kuta (15) - Dives - High Season	2,055	$63	8	17,044	$1,066,169
			Sanur, Kuta (15) - Dives - Low Season	1,590	$63	6	9,891	$618,689
			Padang Bai (5) - Dives - High Season	685	$58	8	5,681	$328,052
			Padang Bai (5) - Dives - Low Season	530	$58	6	3,297	$190,366
			E. Bali (15) - Dives - High Season	450	$58	8	3,732	$215,509
			E. Bali (15) - Dives - Low Season	315	$58	6	1,959	$113,142
	Komodo	Internet; Surveys; Assistance from Komodo Manta Project (44 operators, 5 dive sites); Note: 17 operators work in Komodo and Raja	Op. 1 - Dives	100	$125	6	581	$72,706
			Op. 2 - Dives	24	$119	13	314	$37,294
			Op. 3 - Dives	36	$139	12	418	$58,113
			Op. 4 - Dives	42	$98	10	427	$41,936
			Op. 5 - Dives	46	$79	12	534	$42,005
			Op. 6 - Dives	78	$64	10	793	$50,516
			Op. 7 - Dives	66	$71	4	287	$20,345
			Op. 8 - Dives	111	$101	17	1,933	$194,364
			Op. 9 - Dives	20	$133	6	116	$15,443
			Op. 10 - Dives	12	$118	6	70	$8,245
			Op. 11 - Dives	33	$118	13	431	$50,706
			Op. 12 - Dives	51	$57	4	222	$12,632
			Op. 13 - Dives	48	$47	9	418	$19,699
			Op. 14 - Dives	68	$83	12	790	$65,453
			Op. 15 - Dives	54	$105	12	627	$65,959
			Op. 16 - Dives	120	$70	6	746	$52,372
			Op. 17 - Dives	78	$70	7	566	$39,893
			Op. 18 - Dives	15	$108	6	87	$9,448
			Op. 19 - Dives	44	$101	6	255	$25,781
			Op. 20 - Dives	72	$88	11	784	$69,257
			Op. 21 - Dives	138	$143	9	1,202	$172,295
			Op. 22 - Dives	48	$79	7	348	$27,651

Manta Ray Watching Tourism Extent and Dive Expenditure Estimates (US$)

		Ampat	Op. 23 - Dives	44	$85	9	383	$32,703
			Op. 24 - Dives	56	$91	12	650	$59,128
			Op. 25 - Dives	87	$63	4	379	$23,817
			Op. 26 - Dives	54	$67	10	549	$36,937
			Op. 27 - Dives	28	$56	12	325	$18,159
			Op. 28 - Dives	33	$68	12	383	$25,913
			Op. 29 - Dives	66	$85	12	814	$68,826
			Op. 30 - Dives	93	$124	15	1,350	$167,980
			Op 31 - Dives	32	$132	12	372	$49,208
			Op 32 - Dives	99	$60	9	862	$52,087
Indonesia			Op. 33 - Dives	48	$64	6	279	$17,775
			Op. 34 - Dives	49	$47	9	427	$20,234
			Land based boats - High Season (10)	720	$82	10	7,465	$610,608
			Land based boats - Low Season (2.5)	50	$82	7	363	$29,682
			Op. 1 - Dives	16	$116	13	209	$24,332
			Op. 2 - Dives	26	$96	7	189	$18,159
			Op. 3 - Dives	27	$151	12	314	$47,422
			Op. 4 - Dives	36	$141	17	597	$84,288
			Op. 5 - Dives	26	$90	10	264	$23,868
			Op. 6 - Dives	12	$98	9	105	$10,264
			Op. 7 - Dives	2	$114	12	23	$2,639
			Op. 8 - Dives	8	$110	9	70	$7,636
			Op. 9 - Dives	16	$168	6	93	$15,644
			Op. 10 - Dives	36	$172	6	209	$36,053
			Op. 11 - Dives	24	$142	13	314	$44,528
			Op. 12 - Dives	14	$67	9	122	$8,212
			Op. 13 - Dives	26	$98	12	302	$29,699
			Op. 14 - Dives	34	$102	6	197	$20,144
			Op. 15 - Dives	28	$79	11	305	$23,988
			Op. 16 - Dives	4	$189	9	35	$6,571
			Op. 17 - Dives	24	$124	12	279	$34,445
			Op. 18 - Dives	12	$144	12	139	$19,999
		Internet; Surveys; Assistance from Misool Manta Project and Misool Eco	Op. 19 - Dives	32	$87	12	372	$32,157
	Raja Ampat		Op. 20 - Dives	22	$68	7	160	$10,798

Manta Ray Watching Tourism Extent and Dive Expenditure Estimates (US$)

		and Misool Eco Resort (39 operators, 3 dive sites)	Op. 21 - Dives	26	$140	12	302	$42,279
			Op. 22 - Dives	24	$120	12	279	$33,524
			Op. 23 - Dives	56	$80	7	366	$29,101
			Op. 24 - Dives	50	$103	7	363	$37,345
			Op. 25 - Dives	54	$114	12	666	$76,204
			Op. 26 - Dives	38	$81	10	386	$31,153
			Op. 27 - Dives	16	$157	12	186	$29,178
			Op. 28 - Dives	38	$91	7	276	$24,969
			Op. 29 - Dives	26	$107	12	302	$32,384
			Op. 30 - Dives	12	$76	9	105	$7,967
			Op. 31 - Dives	8	$75	6	46	$3,486
			Op. 32 - Dives	98	$185	20	1,949	$359,785
			Op. 33 - Dives	60	$335	11	646	$216,035
			Op. 34 - Dives	100	$115	15	1,538	$177,423
			Op. 35 - Dives	150	$137	15	2,307	$316,099
			Op. 36 - Dives	100	$162	9	923	$149,892
			Op. 37 - Dives	100	$108	15	1,538	$165,697
			Op. 38 - Dives	100	$154	9	923	$141,939
			Op. 39 - Dives	100	$191	9	923	$175,894
	Sangalaki	Internet; Surveys (7operators, 2 dive sites)	Op. 1 - Dives	60	$94	16	958	$89,885
			Op. 2 - Dives	32	$109	12	372	$40,477
			Op. 3 - Dives	135	$125	25	3,331	$416,740
			Op. 4 - Dives	135	$112	32	4,311	$483,305
			Op. 5 - Dives	135	$130	23	3,135	$407,311
			Op. 6 - Dives	90	$96	36	3,266	$313,161
			Op. 7 - Dives	45	$70	6	261	$18,228
**Indonesia Subtotal**							**139,594**	**$10,655,022**
Japan	Ishigaki Island	Tom Kashiwagi, Takashi Ito, pers. comm. (200 operators, 3 dive sites)	Dive -3 Tank (90%)	42,991	$76		128,974	$9,861,373
			Dive -2 Tank (10%)	4,777	$76		9,554	$730,472
			Snorkels	5,910	$127		5,910	$753,135
	Kuroshima Island		Dive -3 Tank (90%)	4,299	$76		12,897	$986,137
			Dive -2 Tank (10%)	478	$76		955	$73,047
			Snorkels	591	$127		591	$75,313
	Iriomote Islands		Dive -3 Tank (90%)	4,299	$76		12,897	$986,137
			Dive -2 Tank (10%)	478	$76		955	$73,047
			Snorkels	591	$127		591	$75,313

Manta Ray Watching Tourism Extent and Dive Expenditure Estimates (US$)

	Weather Deduction		Total Est. Dives missed		$79		-28,167	-$2,213,873
**Japan Subtotal**							**145,158**	**$11,400,103**
Kiribati	Christmas Island	Internet; Surveys (1 operator, 2 dive sites)	Op. 1 - Dives	50	$50	7	350	$17,500
**Kiribati Subtotal**							**350**	**$17,500**
Madagascar	Nosy Be	Internet; Surveys (11 operators, 1 dive site)	Op. 1 - Dives	78	$39	6	468	$18,252
			Op. 2 - Dives	36	$37	4	144	$5,328
			Op. 3 - Dives	75	$38	3	225	$8,550
			Op. 4 - Dives	66	$38	36	2,376	$90,288
			Ops. 5 - 11 - Dives	511	$38	4	2,213	$84,080
**Madagascar Subtotal**							**5,426**	**$206,498**
Maldives		Anderson *et al.*, 2011 (91 dive sites)	NA	NA	$52	NA	157,000	$8,100,000
**Maldives Subtotal**							**157,000**	**$8,100,000**
Mexico	Socorro (Revillagigedos Islands)	Internet; Surveys (5 operators, 4 dive sites)	Op. 1 - Dives	300	$130	24	7,200	$938,880
			Op. 2 - Dives	440	$130	22	9,680	$1,258,400
			Op. 3 - Dives	340	$128	22	7,480	$955,944
			Op. 4 - Dives	440	$117	18	7,920	$925,056
			Op. 5 - Dives	420	$120	20	8,400	$1,006,320
**Mexico Subtotal**							**40,680**	**$5,084,600**
Mozambique	Inhambane	Internet; Surveys (8 operators, 10 dive sites)	Op. 1 - Dives	1,488	$75	10	14,943	$1,114,585
				1,917	$75	8	15,401	$1,148,742
			Op. 2 - Dives	744	$48	15	11,207	$532,981
				852	$48	10	8,556	$406,900
			Op. 3 - Dives	248	$75	7	1,743	$130,035
				213	$75	5	1,070	$79,774
			Op. 4 - Dives	744	$52	15	11,207	$577,864
				852	$52	12	10,267	$534,538
			Op. 5 - Dives	1,488	$56	14	20,920	$1,172,933
				1,917	$56	8	15,401	$863,484
			Op. 6 - Dives	744	$64	6	4,483	$287,249
				852	$64	4	3,422	$219,298
			Op. 7 - Dives	248	$48	8	1,992	$95,750

Manta Ray Watching Tourism Extent and Dive Expenditure Estimates (US$)

			Op. 7 - Dives	213	$48	5	1,070	$51,398
			Op. 8 - Dives	248	$48	12	2,989	$143,624
				213	$48	8	1,711	$82,237
	Zavora	Internet; Surveys (1 operator, 7 dive sites)	Op. 1 - Dives	112	$52	5	560	$29,120
	Vilanculos	Internet; Surveys (3 operators, 1 dive site)	Op. 1 - Dives	120	$84	6	720	$60,768
			Op. 2 - Dives	120	$79	6	720	$56,700
			Op. 3 - Dives	120	$73	6	720	$52,373
**Mozambique Subtotal**							**129,102**	**$7,640,351**
Myanmar (Currently all perators are based out of Thailand; Myanmar receives a fee from each diver)		Internet; Surveys; Assistance from dive booking agency (10 operators, 3 dive sites)	Op. 1 - Dives	14	$78	13	176	$13,794
			Op. 2 - Dives	12	$61	20	244	$14,772
			Op. 3 - Dives	20	$67	15	294	$19,583
			Op. 4 - Dives	8	$108	10	78	$8,503
			Op. 5 - Dives	8	$132	14	112	$14,749
			Op. 6 - Dives	20	$78	17	336	$26,200
			Op. 7 - Dives	20	$45	10	196	$8,835
			Op. 8 - Dives	12	$131	14	168	$22,070
			Op. 9 - Dives	12	$47	16	192	$8,961
			Op. 10 - Dives (Trip A, B, C)	2	$92	8	17	$1,548
				27	$54	8	227	$12,305
				14	$53	8	118	$6,286
**Myanmar Subtotal**							**2,158**	**$157,606**
New Caledonia		Internet; Surveys (10 operators, 2 dive sites)	Op. 1 - Dives	68	$102	11	748	$75,922
			Op. 2 - Dives	68	$102	6	408	$41,412
			Op. 3 - Dives	68	$102	11	748	$75,922
			Op. 4 - Dives	68	$111	11	748	$83,260
			Op. 5 - 10 - Dives	408	$102	6	2,448	$248,472
**New Caledonia Subtotal**							**5,100**	**$524,988**
			Op. 1 - Dives	88	$65	44	3,872	$251,680
				138	$65	16	2,208	$143,520
			Op. 2 - Dives	180	$80	54	9,720	$777,600
				52	$80	24	1,248	$99,840
			Op. 3 - Dives	104	$65	10	1,040	$67,600
			Op. 4 - Dives	104	$65	10	1,040	$67,600

Manta Ray Watching Tourism Extent and Dive Expenditure Estimates (US$)

Palau		Internet; Surveys; Assistance from PalauMantaRays.com (17 operators, 2 dive sites)	Op. 5 - Dives	156	$65	12.8	1,997	$129,792
			Op. 6 - Dives	104	$60	10	1,040	$62,400
			Op. 7 - Dives	104	$60	10	1,040	$62,400
			Op. 8 - Dives	104	$60	10	1,040	$62,400
			Op. 9 - Dives	104	$60	10	1,040	$62,400
			Op. 10 - Dives	104	$60	10	1,040	$62,400
			Op. 11 - Dives	104	$60	10	1,040	$62,400
			Op. 12 - 14 - Dives	312	$65	10	3,120	$202,800
			Op. 15 - Dives	125	$69	14	1,688	$117,067
			Op. 16 - Dives	115	$69	12	1,380	$95,735
			Op. 17 - Dives	88	$69	21	1,838	$127,473
**Palau Subtotal**							**35,390**	**$2,455,108**
Philippines	Ticao	Internet; Surveys (4 Operators, 4 dive sites)	Op. 1-4 - Dives	90	$27	10	900	$24,300
				90	$27	45	4,050	$109,350
				60	$27	5	300	$8,100
	Malapascua	Internet; Surveys (20 operators, 1 dive site)	Op. 1 - Dives	156	$27	15	2,340	$63,180
				156	$27	6	936	$25,272
			Op. 2 - Dives	55	$27	14	770	$20,790
				26	$27	14	364	$9,828
			Op. 3 - 20 - Dives	182	$27	20	3,640	$98,280
				182	$27	7	1,214	$32,776
	Tubbataha	Internet; Surveys (5 operators, 3 dive sites)	Op. 1 - Dives	35	$113	16	560	$63,115
			Op. 2 - Dives	35	$113	23	788	$88,827
			Op. 3 - Dives	51	$121	18	918	$111,471
			Op. 4 - Dives	51	$122	15	765	$93,439
			Op. 5 - Dives	51	$125	18	918	$114,750
**Philippines Subtotal**							**18,463**	**$863,479**
Papua New Guinea	Milne Bay (Gonubalabala)	Internet; Surveys (5 operators, 2 dive sites)	Op. 1 - Dives	5	$69	10	48	$3,333
			Op. 2 - Dives	5	$97	13	64	$6,222
			Op. 3 - Dives	110	$86	13	1,408	$121,049
			Op. 4 - Dives	45	$89	10	432	$38,640
			Op. 5 - Dives	10	$105	6	60	$6,317
**Papua New Guinea Subtotal**							**2,012**	**$175,561**
Solomon		Internet; Surveys (4 [illegible]	Op. 1 - Dives	59	$136	12	708	$96,288
			Op. 2 - Dives	100	$136	10	1,000	$136,000

Manta Ray Watching Tourism Extent and Dive Expenditure Estimates (US$)

Islands		operators, 5 dive sites)	Op. 3 - Dives	50	$78	16	800	$62,176
			Op. 4 - Dives	100	$62	4	400	$24,868
**Solomon Islands Subtotal**							**2,908**	**$319,332**
Sudan		Internet; Surveys (7 operators, 1 dive site)	Op. 1 - Dives	2	$77	13	25	$1,935
			Op. 2 - Dives	2	$77	13	25	$1,934
			Op. 3 - Dives	2	$83	15	31	$2,568
			Op. 4 - Dives	2	$55	11	22	$1,236
			Op. 5 - Dives	2	$85	18	36	$3,105
			Op. 6 - Dives	2	$65	17	34	$2,198
			Op. 7 - Dives	1	$77	12	7	$530
**Sudan Subtotal**							**181**	**$13,506**
			Op. 1 - Dives	38	$44	13	479	$21,006
			Op. 2 - Dives	124	$60	11	1,389	$82,929
			Op. 3 - Dives	125	$48	14	1,750	$84,763
			Op. 4 - Dives	125	$48	10	1,225	$59,334
			Op. 5 - Dives	124	$38	20	2,517	$96,462
			Op. 6 - Dives	108	$82	13	1,361	$111,088
				108	$82	10	1,058	$86,402
			Op. 7 - Dives	144	$42	11	1,613	$67,589
			Op. 8 - Dives	105	$48	20	2,058	$97,902
			Op. 9 - Dives	24	$73	10	235	$17,266
			Op. 10 - Dives	75	$44	13	945	$41,343
			Op. 11 - Dives	81	$93	15	1,191	$111,089
				15	$80	15	221	$17,727
			Op. 12 - Dives	100	$82	11	1,050	$85,823
				8	$138	14	112	$15,451
			Op. 13 - Dives	24	$47	10	235	$11,025
			Op. 14 - Dives	88	$66	14	1,232	$81,582
			Op. 15 - Dives	120	$37	23	2,736	$100,436
			Op. 16 - Dives	80	$43	10	784	$33,609
			Op. 17 - Dives	105	$52	13	1,323	$68,318
			Op. 18 - Dives	130	$81	8	1,092	$88,311
			Op. 19 - Dives	56	$69	8	470	$32,447
			Op. 20 - Dives	140	$54	17	2,352	$127,738
			Op. 21 - Dives	112	$54	13	1,411	$76,491
			Op. 22 - Dives	108	$42	11	1,210	$50,795

Manta Ray Watching Tourism Extent and Dive Expenditure Estimates (US$)

Thailand	Similan Surin Islands	Internet; Surveys; Assistance from dive booking agency (62 operators, 2 dive sites); Note: 2 operators work in both locations, Thailand total = 76	Op. 23 - Dives	140	$54	10	1,372	$73,663
			Op. 24 - Dives	124	$60	10	1,215	$73,304
			Op. 25 - Dives	164	$46	20	3,355	$155,062
				160	$46	17	2,678	$123,774
				159	$52	17	2,662	$139,432
			Op. 26 - Dives	75	$61	10	735	$44,545
			Op. 27 - Dives	108	$71	13	1,361	$97,206
			Op. 28 - Dives	225	$42	17	3,780	$157,556
			Op. 29 - Dives	96	$42	6	538	$22,449
			Op. 30 - Dives	92	$76	10	902	$68,624
			Op. 31 - Dives	136	$63	13	1,741	$110,416
			Op. 32 - Dives	120	$37	17	2,016	$74,015
			Op. 33 - Dives	24	$90	14	336	$30,139
			Op. 34 - Dives	136	$63	14	1,904	$120,767
			Op. 35 - Dives	80	$56	15	1,232	$69,492
			Op. 36 - Dives	152	$53	17	2,554	$136,229
			Op. 37 - Dives	144	$82	8	1,210	$99,740
				144	$82	14	2,016	$166,233
				108	$66	17	1,814	$120,309
			Op. 38 - Dives	112	$116	9	1,019	$118,477
			Op. 39 - Dives	120	$37	14	1,728	$63,433
			Op. 40 - Dives	182	$44	20	3,567	$156,875
				184	$38	20	3,606	$138,138
			Op. 41 - Dives	210	$35	18	3,822	$134,848
			Op. 42 - Dives	64	$53	8	538	$28,364
			Op. 43 - Dives	40	$134	11	448	$60,243
			Op. 44 - Dives	124	$76	13	1,562	$119,039
			Op. 45 - Dives	180	$51	13	2,268	$116,782
			Op. 46 - Dives	180	$43	11	2,016	$87,000
			Op. 47 - Dives	108	$58	17	1,814	$104,498
			Op. 48 - Dives	141	$74	13	1,777	$132,181
				8	$66	18	144	$9,523
			Op. 49 - Dives	104	$109	10	997	$108,875
			Op. 50 - Dives	52	$116	7	356	$41,331
			Op. 50 - Snorkels	52	$59	7	356	$20,993
			Op. 51 - Dives	104	$111	7	748	$82,959

Manta Ray Watching Tourism Extent and Dive Expenditure Estimates (US$)

			Op. 52 - Dives	104	$84	18	1,869	$157,449
			Op. 53 - Dives	156	$76	10	1,495	$114,087
			Op. 54 - Dives	104	$83	7	748	$62,111
			Op. 55 - Dives	104	$96	10	997	$95,555
			Op. 56 - Dives	260	$95	10	2,492	$236,282
			Op. 57 - Dives	52	$98	10	498	$48,646
			Op. 58 - Dives	52	$98	9	490	$47,778
			Op. 59 - Dives	104	$103	10	997	$103,084
			Op. 60 - Dives	104	$90	10	997	$89,764
			Op. 61 - Dives	104	$83	17	1,745	$144,925
			Op. 62 - Dives	52	$98	10	534	$52,431
	Phuket - Racha Noi	Internet; Surveys (16 operators, 1 dive site)	Op. 1 - Dives	2	$142	15	30	$4,270
			Op. 2 - Dives	23	$73	10	225	$16,476
			Op. 3 - Dives	96	$58	4	403	$23,495
			Op. 4 - Dives	52	$55	23	1,202	$65,822
			Op. 5 - Dives	52	$55	23	1,202	$65,822
			Op. 6 - Dives	52	$66	10	534	$35,214
			Op. 7 - Dives	26	$80	15	400	$32,039
			Op. 8 - Dives	26	$66	13	334	$22,009
			Op. 9 - Dives	208	$76	15	3,051	$230,909
			Op. 10 - Dives	52	$80	8	427	$34,175
			Op. 11 - Dives	195	$64	13	2,503	$160,193
				130	$53	15	2,002	$106,767
				104	$53	18	1,869	$99,649
			Op. 12 - Dives	104	$66	13	1,335	$88,034
			Op. 13 - Dives	156	$65	8	1,282	$83,127
			Op. 14 - Dives	52	$82	10	534	$43,873
			Op. 15 - Dives	78	$58	13	1,001	$57,727
			Op. 16 - Dives	26	$71	13	334	$23,596
**Thailand Subtotal**							**121,767**	**$7,418,750**
United States	Kona, Hawaii	Manta Pacific Research Foundation, unpubished data (24 operators, 1 dive site)	Op. 1 - 24 - Dives	NA	$122		17,095	$2,092,862
			Op. 1 - 24 - Snorkels	NA	$82		17,017	$1,402,076
	Maui, Hawaii	Internet; Surveys (12 operators, 2 dive	Op. 1 - Dives	900	$70	18	16,200	$1,134,000
			Op. 2 - Dives	50	$55	7	350	$19,250

Manta Ray Watching Tourism Extent and Dive Expenditure Estimates (US$)

		sites)	Op. 3 - 10 - Dives	25	$55	10	250	$13,750
**United States Subtotal**							**50,912**	**$4,661,938**
**Global Totals**		**190 Manta Dive Sites**					**1,037,703**	**$73,347,286**
