# Supplementary material for: The Global Economic Impact of Manta Ray Watching Tourism
Source: PLoS One. 2013 May 31;8(5):e65051. doi: 10.1371/journal.pone.0065051 (PMC3669133; doi:10.1371/journal.pone.0065051)
Supplement: Table S2 — Direct Economic Impact of Manta Ray Watching Tourism. Research results used to calculate ratios of total expenditures to dive only expenditures to produce the country direct economic impact estimates summarized in Table 3. (PDF) [file pone.0065051.s002.pdf]

Table S2. Direct Economic Impact of Manta Ray Watching Tourism (US$)

Expenditure Ratios from Diving Expenditure Sources	Local Expenditure / Participant	Dive Expenditure	Total Local Exp. / Dive Exp. (DEI Ratio)	Trip Type	Ref Year	Source		
Australia, GBR (N. Queensland) - Per Trip	AUD 2,600	AUD 1,300	200%	Live aboard - varied	2006-7	[42]		
W. Australia (Ningaloo) - Per Day	AUD 186	AUD 76	246%	Whale sharks	2006	[43]		
Fiji - Per Day	USD 212	USD 84	252%	Sharks	2011	[18]		
Fr. Polynesia (Moorea) - Per Day	USD 377	USD 139	271%	Lemon sharks	2011	[6]		
Palau - Per Trip	USD 2,081	USD 749	278%	Sharks	2010	[50]		
Thailand - Per Trip	THB 40,000	THB 24,010	167%	Diving	2007-9	[49]		

Expenditure Ratios from Whale Watching Expenditure Sources	WW Ticket Exp. / Participant	Assoc. Exp. / Participant WW	Total Local Exp. / Participant WW	Manta Dive Trip Cost / Participant	Total Exp. /Participant Manta Diving	Total Exp. / WW Ticket Cost (DEI Ratio WW)	Total Exp. / MD Cost (DEI Ratio Manta Diving)	Ref Year / Source
Australia, GBR (Queensland)	$28	$146	$174	$108	$254	616%	235%	[20], 2008
W. Australia	$34	$156	$191	$108	$264	554%	245%	[20], 2008
Costa Rica	$50	$150	$200	$100	$250	398%	250%	[20], 2008
Ecuador	$1,171	$234	$1,405	$150	$384	120%	256%	[20], 2008
FSM	$46	$111	$157	$120	$231	343%	192%	[20], 2008
Fr. Polynesia (Bora Bora)	$94	$121	$215	$137	$258	229%	188%	[20], 2008
Indonesia	$14	$33	$47	$78	$111	332%	142%	[20], 2008

Direct Economic Impact of Manta Ray Watching Tourism (US$)

Japan	$38	$80	$118	$211	$330	308%	156%	[20], 2008
Madagascar	$51	$63	$115	$76	$139	224%	184%	[20], 2008
Maldives	$36	$40	$76	$44	$84	211%	191%	[20], 2008
Mexico	$53	$450	$503	$446	$896	942%	201%	[20], 2008
Mozambique	$58	$85	$143	$118	$203	246%	171%	[20], 2008
New Caledonia	$85	$125	$210	$103	$229	247%	221%	[20], 2008
Philippines	$32	$60	$92	$94	$154	289%	164%	[20], 2008
Sudan (Egypt #'s)	$58	$98	$156	$142	$240	269%	169%	[20], 2008
USA, Hawaii	$38	$254	$292	$110	$364	778%	331%	[20], 2008

Country	Manta Dive Expenditure	Trip Exp / Dive Exp	Direct Economic Impact	Source(s)
Australia	$6,529,435	223%	$14,560,640	[42,43]
Costa Rica	$109,200	250%	$273,000	[20]
Ecuador	$726,126	256%	$1,858,883	[20]
Fiji	$630,148	252%	$1,587,973	[18]
Fr. Polynesia	$1,367,625	271%	$3,706,264	[6]
FSM	$4,091,520	192%	$7,855,718	[20]
India	$198,890	167%	$332,146	[49]
Indonesia	$10,655,022	142%	$15,130,131	[20]
Japan	$11,400,103	156%	$17,784,161	[20]
Kiribati	$17,500	192%	$33,600	[20] - FSM
Madagascar	$206,498	184%	$379,956	[20]
Maldives	$8,100,000	191%	$15,471,000	[20]
Mexico	$5,084,600	201%	$10,220,046	[20]
Mozambique	$7,640,351	171%	$13,065,000	[20]
Myanmar	$157,606	167%	$263,202	[49]
New Caledonia	$524,988	221%	$1,160,223	[20]
Palau	$2,455,108	278%	$6,825,200	[50]
Papua New Guinea	$175,561	192%	$337,077	[20] - FSM
Philippines	$863,479	164%	$1,416,106	[20]
Solomon Islands	$319,332	192%	$613,117	[20] - FSM

Direct Economic Impact of Manta Ray Watching Tourism (US$)

Sudan	$13,506	169%	$22,825	[20] - Egypt
Thailand	$7,418,750	167%	$12,389,313	[49]
USA	$4,661,938	331%	$15,431,015	[20]
**Global Total**	**$73,347,286**		**$140,716,597**	

DEI Ratio Notes

Summary:

13 - Ratios adapted from O'Connor et al. [20] replacing WW ticket expenditure (DE) with Manta dive trip cost:
Costa Rica, Ecuador, FSM, Indonesia, Japan, Madagascar, Maldives, Mexico, Mozambique, New Caledonia, Philippines, Sudan (using #'s from Egypt), USA (Hawaii)

3 - Applied FSM ratio from [20]: Solomon Islands, Papua New Guinea and Kiribati

4 - Ratios from published studies on economic impact of shark diving:
Australia [42,43], Fiji [18], French Polynesia [6], Palau [50]

3 - Ratio from published study by Tourism Authority Thailand[49] on scuba diving tourists and expenditures:
Thailand and applied same ratio to Myanmar and India, since trips to these locations originate from Thailand on Thai boats

Australia: There were two published expenditure estimates for diving - Jones et al. [43] for Western Australia; Stoeckl et al. [42] for GBR (Queensland); and two estimates for whale watching expenditures from O'Connor et al. [20] - one for Western Australia and one for GBR (Queensland).

Stoeckl ratio: Median cost of live aboard dive trip ~ AUD 1,300; Median regional assoc. exp./diver ~ AUD 1,300

Jones ratio: Whale shark tourism participants - Mean expenditure / day; Mean activity expenditure / day

We opted to use the average of the 2 shark diving expenditure estimates.

The four Australia estimates were:

	Queensland	W. Australia	Avg Australia
O'Connor [20]	235%		240%
O'Connor [20]		245%	
Stoeckl [42]	200%		**223%**
Jones [43]		246%	

Fiji: Average daily local expenditures by divers in Fiji per Vianna et al. [18];
Average daily cost for manta diving per this study, since dive costs were not available.

Direct Economic Impact of Manta Ray Watching Tourism (US$)

**French Polynesia:** Ratio estimated from the following values provided in Clua et al. [6]:
Avg. Daily Associated Expenditures: ($186 acc. + $52 meals)= $ 238 (AE)
Avg. Cost (Int'l and Local divers) of 2 tank dive trip = $139 (DE)
Dive expenditure plus associated expenditures = $377
**Palau:** From Vianna et al. [50], avg. trip expenditure per diver; avg. dive expenditure per diver per trip
**Thailand:** From Tourism Authority Thailand [49], avg. trip expenditure per diver; avg. dive expenditure per diver per trip
