# Supplementary material for: The Global Economic Impact of Manta Ray Watching Tourism
Source: PLoS One. 2013 May 31;8(5):e65051. doi: 10.1371/journal.pone.0065051 (PMC3669133; doi:10.1371/journal.pone.0065051)
Supplement: File S1 — Dive Operator Surveys. Survey questions sent to live aboard dive operators and land-based dive operators. (PDF) [file pone.0065051.s003.pdf]

File S1. Dive Operator Surveys

Live aboard dive operators:

1. Are manta dive sites (sites where manta rays are a primary attraction) a regular part of your itinerary in [location]?

2. If so, how many total dives are generally included in a trip and how many of the dives are at manta dive sites?

3. Are the mantas seasonal? If so, how long is the season when you can take divers to this site(s) to see mantas?

4. On average how many divers do you take per trip in [location]?

5. What is the cost per person for a trip? For how many days?

6. How many other boats/operators also visit this / these manta dive site(s)?

7. Where do you think mantas rank among sea life divers most want to see?

8. Do you think manta rays are an important reason divers come to [location]? Do you think they're important to your business?

Please provide any other comments you'd like to add that might be helpful with assessing the value of manta rays to your business and to the local community overall.

Notes: Almost all the live aboard dive operations and/or dive travel agencies that specialize in booking live aboard dive trips provide very detailed information on their web sites, including trip schedules, pricing, maximum number of divers per trip, as well as dive sites visited with highlights for each site and often detailed trip itineraries.

Land based dive operators:

1. Do you offer manta ray dive or snorkel trips (trips to dive sites where manta rays are a primary attraction)?

2. Are mantas seen all year? If only seasonal, what are the months when mantas are present?

3. Including all boats in your operation, how many trips on average does [dive operation] make to manta ray dive sites each week? (Considering the average number of trips cancelled due to weather or other reasons)

4. On average how many divers or snorkelers (from [dive operation]) are on these dives?

5. What is the cost per diver /snorkeler (please specify one dive or two dive trip, etc)?

6. Do you know how many other dive companies also take divers / snorkelers to this (these) site(s)?

7. Where do you think manta rays rank among sea life that divers most want to see?

8. Do you think manta rays are an important reason that divers visit [location]? Are they important to your business?

Please provide any other comments you'd like to add that might be helpful with assessing the value of manta rays to your business and to the local community overall.

Notes: In some locations, some of the small dive operators do not have web sites, but other area operators were generally helpful with providing information on the total number of operators in the area and the estimated number of divers / snorkelers the other operators take to the manta dive sites. While the web sites for land based operators were generally not as detailed as those for the live aboard companies, most of them include information on the number of boats they have (often with pictures of their boats), the maximum number of divers they take per trip, dive costs, dive sites visited and highlights for each site and the best times of year to see popular animals, such as manta rays. Some also included detailed weekly dive schedules.
